# Syndecans in Inflammation at a Glance

**DOI:** 10.3389/fimmu.2020.00227

**Published:** 2020-02-18

**Authors:** Sandeep Gopal

**Affiliations:** Development and Stem Cells Program, Department of Anatomy and Developmental Biology, Monash Biomedicine Discovery Institute, Monash University, Melbourne, VIC, Australia

**Keywords:** syndecan, proteoglycan, extravasation, inflammation, shedding, cytokines, chemokine gradient

## Abstract

Syndecans are transmembrane proteoglycans with heparan and chondroitin sulfate chains attached to their extracellular domain. Like many proteoglycans, they interact with a large number of ligands, such as growth factors, adhesion receptors, soluble small molecules, proteinases, and other extracellular matrix proteins to initiate downstream signaling pathways. Syndecans play a major role in inflammation, mainly by regulating leukocyte extravasation and cytokine function. At the same time, syndecans can undergo cytokine mediated changes in their expression levels during inflammation. The function of syndecans during inflammation appears to depend on the stage of inflammation, sulfation of heparan/chondroitin sulfate chains, the rate of ectodomain shedding and the solubility of the ectodomains. From the current literature, it is clear that syndecans are not only involved in the initial recruitment of pro-inflammatory molecules but also in establishing a balanced progression of inflammation. This review will summarize how cell surface and soluble syndecans regulate multiple aspects of inflammation.

## Introduction

Inflammation is the immediate response of the body to combat an infection or injury. It is a cascade of complex immunological events resulting from the disruption of tissue homeostasis, which acts to remove the source of infection or restore damaged tissue. When left unchecked, inflammation will result in further tissue damage and injury (1). Inflammation can either be acute or chronic. Acute inflammation (e.g., wounding) is a controlled short-term process that results in the healing of the damaged tissue or the removal of infection, whereas chronic inflammation (e.g., cancers) is a persistent response that leads to further tissue damage. Chronic inflammation often does not present visible cardinal signs of inflammation, such as redness (rubor), increased heat (calor), swelling (tumor), pain (dolor), or loss of function (functio laesa) (2). However, it can lead to more serious conditions, such as fibrosis and cancers (3, 4). Inflammation is associated with modifications to the local vasculature and elevated blood flow that permits the recruitment of leukocytes, plasma proteins, and soluble molecules to the site of inflammation (5). Key steps in inflammation involve recognition of the inducers, signal transduction, release of pro-inflammatory molecules, activation of the effectors of inflammation, and resolution of the inflammation (6). The inducers are recognized by a range of receptors, such as toll-like receptors and nucleotide-binding domain and leucine-rich-repeat-containing receptors, leading to the activation and nuclear translocation of the transcription factor NF-κB (7–9). This induces the expression of a number of pro-inflammatory cytokines, such as interleukin-1β (IL-1β), IL-6, IL-8, IL-12, and Tumor Necrosis Factor-α (TNF-α). While several molecules are involved in inflammation, cytokines play a central role as both pro-inflammatory and anti-inflammatory molecules (6). However, the classification of pro-inflammatory and anti-inflammatory is not absolute, as some of the cytokines are known to play both roles (10). In the next stage, effector cells, such as neutrophils and monocytes are recruited to the site of inflammation leading to a process called degranulation (11). Neutrophils expedite the release of reactive oxygen species, reactive nitrogen species, and protein degrading enzymes. This creates a highly toxic environment for pathogens as well as the host tissue, leading to the destruction of both. In the final phase of inflammation, macrophages ensure minimal damage to the host tissue by restricting neutrophil migration while enhancing monocyte recruitment to the site of inflammation (11, 12).

## Syndecans in Inflammation

**Figure 1:**
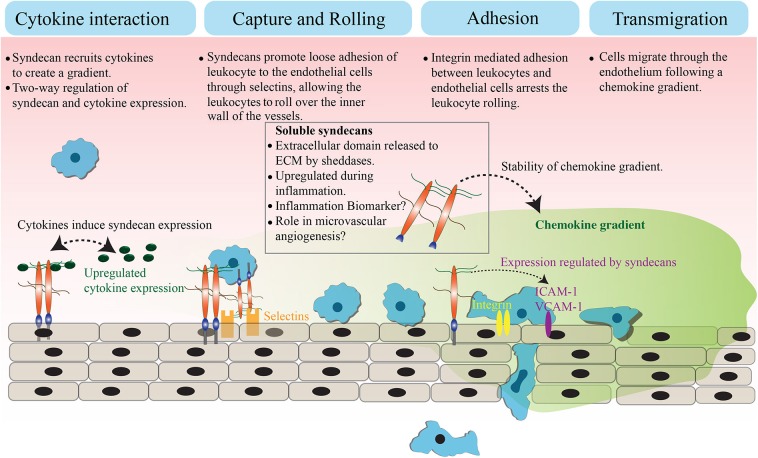
Role of syndecans in extravasation. Syndecans bind to inflammatory cytokines and chemokines through GAG chains. This interaction appears to trigger a feedback signaling, which results in an elevated expression of both syndecan and cytokines. The GAG chain mediated binding of syndecan with chemokine and the subsequent shedding of syndecan-chemokine complex results in a stable chemokine gradient. Syndecans mediate a loose interaction between leukocytes and endothelial cells through selectins, which reduces the pace of leukocyte movement and they bigin to roll over the vessel surface. The adhesion between leukocytes and endothelial cells is strengthened by an integrin controlled signaling and other cell adhesion molecules, leading to the arrest of leukocyte rolling. Attached leukocytes begin transmigration directed by a stable chemokine gradient to the site of inflammation.

Syndecans are transmembrane proteoglycans that can interact with a large number of ligands including growth factors, adhesion receptors, cytokines, chemokines, proteinases, and other extracellular matrix proteins (13). As a result of these ligand interactions, syndecans initiate a number of biological signaling events relevant to cell adhesion, angiogenesis, inflammation, and tissue repair (14–18). The mammalian genome encodes four syndecans; syndecan−1, −2, −3, and −4. Syndecans not only maintain cell homeostasis under normal conditions but also regulate inflammatory responses during infection and trauma (Figure 1). Syndecans are one of the major sources of glycosaminoglycan (GAG) chains on the cell surface. They control a large number of cytokines though GAG chain mediated binding to stimulate inflammatory response (19–21). While syndecan-1 is the most studied syndecan in the context of inflammation, other syndecans are also shown to have roles in inflammatory response in various models. Syndecan-1 and -4 knockout mouse models have provided a great deal of information about the role of syndecans in inflammation, where they appear to be involved in multiple aspects of inflammation from leukocyte recruitment through to the resolution of inflammation (22, 23). One common observation during inflammation is that the total expression of syndecans at protein level can be significantly upregulated (21, 24). For instance, the levels of syndecan-1 and syndecan-4 are elevated during myocardial injury. Similarly, syndecan-2 expression is upregulated in endothelial cells, fibroblasts and intestinal epithelial cells by inflammatory stimuli, such as TNFα or hypoxia (25, 26). It has been previously reported that the level of syndecans in the serum of animals is directly related to the severity of the inflammation (27, 28). Other reports suggest that the loss of syndecans from cells, possibly by shedding, regulates inflammatory responses (28). This review will discuss the role of cell surface and soluble syndecans in cytokine regulation and leukocyte extravasation with a focus on syndecan−1,−2, and−4, while syndecan-3 is discussed in detail in another review of this issue.

During leukocyte extravasation, the leukocytes move out of circulation and adhere in proximity to the sites of inflammation (6). Syndecans, along with glypicans are the major heparan sulfate proteoglycans (HSPG) that control this process (22, 29, 30). HSPGs loosely bind to L- and/or P-selectins on the inner wall of vessels to slow down the flow of leukocytes (31), leading to leukocyte rolling on the surface of the endothelium. This suggests the involvement of HSPGs as pro-inflammatory molecules, specifically to support leukocyte rolling. However, knockout studies showed an elevated inflammatory response in the absence of syndecan-1 or syndecan-4 in mice, indicating that syndecans are inflammation antagonists instead (22, 28, 32). These observations were explained when an increase in leukocyte adhesion and transendothelial migration with prolonged edema formation was identified in syndecan-1 knockout mice subjected to a myocardial infarction (33). Another report suggests that syndecan-1 is essential to limit inflammation and apoptosis during lung injury after an influenza infection (34). Similarly, syndecan-1 is required to maintain the motility of macrophages during chronic inflammation in atherosclerosis, where the absence of syndecan-1 resulted in persistent inflammation (35). In addition to that, syndecan-1 knockout mice exhibit increased expression of pro-inflammatory cytokines (TNFα, IL-6, CCL-5, and CCL-3) and adhesion molecules (ICAM-1 or VCAM-1) during oxazolone-induced delayed-type hypersensitivity and dextran sodium sulfate-induced colitis (36, 37). These data support the idea that syndecan-1 is anti-inflammatory. The negative regulation of strong leukocyte adhesion to endothelium by syndecan-1 is proposed to be the reason behind its anti-inflammatory role. In general, strengthening of leukocyte adhesion to the endothelium is the penultimate step of leukocyte extravasation, which is mediated by integrins and adhesion molecules (e.g., ICAM-1, VCAM-1). It has been suggested that syndecan-1 may oppose integrins, likely by competition for ligands to control strong leukocyte adhesion. Therefore, the absence of syndecan-1 may have promoted integrin mediated leukocyte adhesion to the endothelium (38). Taken together, syndecan-1 appears to support the initial regulation of leukocyte movement whereas it negatively regulates the later leukocyte adhesion and migration. Similar observations were made with syndecan-4, where chemotaxis experiments revealed that syndecan-4 mediates anti-thrombin induced inhibition of leukocyte migration in a heparan sulfate chain-dependent manner (39). These observations indicate that syndecans function to create a balanced inflammatory response by controlling distinct aspects of leukocyte extravasation. In some chronic inflammatory diseases, such as asthma, syndecan-4 also control T-helper 2 (Th2) derived immune response. Asthma is characterized by airway inflammation, which is caused by an inappropriate Th2 cell response. The blocking of syndecan-4 in ovalbumin sensitized mice resulted in a reduced inflammation due to the defective migration of antigen presenting dendritic cells. This affects the Th2 derived immune response where dendritic cells failed to deliver signals to Th2 cells (40).

It is well-known that the balancing of pro-inflammatory and anti-inflammatory molecules dictates the final outcome of an inflammatory response. The increase in leukocyte recruitment to the inflammation site is usually supported by an increase in the expression of cytokines and adhesion molecules. It has been reported that HSPGs can promote or inhibit both pro-inflammatory and anti-inflammatory cytokines by direct binding through their GAG chains (41–43). Depending on the model under investigation, different syndecans have shown to bind either pro-inflammatory or anti-inflammatory cytokines. Syndecans facilitate a gradient of a group of small cytokines, known as chemokines, during leukocyte rolling. For example, the binding of chemokines (e.g., IL-8) to syndecan-1 through GAG chains allows the stabilization of the chemokines on the endothelial surface and creates a tethering effect for the chemokine. The later shedding of the chemokine-Syndecan-1 complex leads to a stable chemokine gradient (44, 45). This chemokine gradient appears to be crucial for maintaining the directionality of leukocyte migration (46). Another report showed that syndecan-1 regulates pro-inflammatory cytokine IL-17 during Psoriasiform Dermatitis, where the absence of syndecan-1 resulted in increased skin inflammation (47). Both syndecan-1 and IL-17 are shown to be increased in nasal polyps, a chronic inflammation in the nasal cavity (48). Syndecan-2 expression is elevated in endothelial cells during inflammation, where they can directly bind with cytokines (e.g., CXCL-8) and induce the expression of other cytokines (e.g., IL-1α, IL-17A^+^) (25, 49). The silencing of syndecan-4 in HUVEC cells resulted in an elevation of pro-inflammatory factors (e.g., CXCL-8), which suggests that a loss of syndecan-4 may result in an aggravated inflammatory response. This appears to agree with syndecan-4 knockout mice studies, where lipopolysaccharide (LPS) induced pulmonary inflammation resulted in increased early neutrophil migration and higher titers of chemokines in bronchoalveolar lavage fluid. In this case, the expression of chemokines was reduced when the bronchial epithelial cells were pre-treated with heparin or syndecan-4 before LPS treatment (50). While syndecans appears to control inflammatory cytokines, the inverse is also possible, with the expression of syndecans being regulated by cytokines. In some cases, the same cytokines even have opposite effects on the expression of different syndecans. For instance, IL-1β and IL-6 treatment downregulated syndecan-1 expression in isolated rat hepatocytes while syndecan-2 expression is upregulated by the same (51). The same study also reported an increase in syndecan-1 expression in response to a TNF-α treatment. Similar observations were made in human periodontal fibroblasts and osteoblasts (52). Growing HUVEC endothelial cells in the presence of inflammatory mediators, such as IL-1β and LPS resulted in a rapid elevation of syndecan-4 expression (53). On the other hand, TNF-α treatment of HT29 cells resulted in the downregulation of syndecan-1 expression (54). These findings clearly indicate a feedback mechanism to control the expression of syndecans during inflammation. Further investigations are required to provide clarity to the effect of this feedback regulation on inflammation.

As syndecans show both pro- and anti-inflammatory roles in knockout models, one might wonder the reason behind this. For instance, an arthritis model generated by injecting CXCL1 into mice knee showed a decreased disease severity in syndecan-3 null mice compared to wildtype, possibly through a reduced neutrophil accumulation and cartilage degradation in null mice (55). However, the administration of CXCL1 into the skin resulted in increased neutrophil recruitment compared to wildtype animals (55). One explanation for this opposite effect could be, that syndecan-3 is promoting pro-inflammatory cytokines in skin and anti-inflammatory cytokines in the cartilage. Another explanation is that the deletion of syndecan-3 resulted in distinct response from other syndecans in each system under evaluation. This study did not examine the expression changes of other syndecans after syndecan-3 deletion. This becomes relevant when the deletion of one syndecan leads to opposite inflammatory responses to same stimulus in different tissues. It is possible that the removal of syndecan-3 may have influenced the expression of different members syndecan family in each system, eventually leading to a compensatory or opposite response. Earlier studies regarding the role of syndecans in inflammation did not take potential compensation between syndecans into account. There is no obvious developmental phenotype for mice lacking syndecan-1 or syndecan-4, which may indicate compensation between syndecans. However, when these mice are injured, they exhibit profound phenotypes. This leads to the question on whether the phenotype observed is a result of the loss of one syndecan or due to the compensatory effect by an alternate syndecan. In addition, each syndecan may be interacting with distinct cytokines and the second syndecan may be signaling through different ligands instead of simply compensating for the deleted syndecan.

Another aspect of syndecan function in inflammation yet to be addressed is their role in matrix turnover and mechanotransduction. ECM remodeling is observed during chronic inflammation, which is usually mediated by matrix degrading enzymes (e.g., Matrix metalloproteinases) (56). Cytokines promote the expression of catabolic enzymes leading to matrix degradation and turnover (57, 58). Since syndecans are one of the key regulators of cytokine function during inflammation, it is safe to assume that syndecans contribute to matrix turnover. ECM remodeling and increased deposition of ECM components are common during the development of fibrosis, a prominent outcome of chronic inflammation (59). Cardiac disease models showed that syndecan-1 mediates fibrosis resulting from an enhanced deposition of collagen (60, 61). Similar observations were made in the lung where syndecan-4 plays an important role in limiting lung fibrosis during inflammation (62, 63). ECM remodeling contributes significantly to the mechanical properties of the matrix. The role of mechanosignaling in leukocyte extravasation has been reported previously (64). While the role of syndecans as mechanosensors is not fully elucidated, they can play a significant role in mechanotransduction. Syndecan mediation of cell-matrix and cell-cell adhesion, and cytoskeleton organization has been extensively studied (13, 14, 65–67). Therefore, it is safe to assume that syndecans may control mechanical forces generated by the adhesion associated molecules during inflammation. A previous report supports this notion, where the syndecan-1 knockout endothelial cells failed to form a phospho-paxillin gradient in response to the shear stress generated by atheroprotective flow. Paxillin is a structural protein in the focal adhesions and the defective phosphorylation of paxillin resulted in a pro-inflammatory phenotype (28). In addition, syndecan-1 and−4 have shown to control the function of stretch-activated calcium channels in epithelial cells and fibroblasts, respectively. This could well be relevant for inflammation, where calcium is not only a known influencer, but also important in conditions, such as wound contraction and shear (68).

## Soluble Syndecans in Inflammation

Syndecans exist either as transmembrane form or as soluble extracellular domains (69). Proteolytic cleavage of syndecan core proteins at the juxtamembrane site is associated with inflammation, mainly as a result of the actions by enzymes called sheddases (e.g., MMPs, ADAMTS) (70). The cleavage of the core protein results in the delivery of extracellular domain to the matrix and then to the circulation. Several cytokines (e.g., IL-8, IL-17, TNFα) can induce core protein cleavage by sheddases, likely by directly interacting with GAG chains (45). The cleaved core proteins, carrying glycosaminoglycan chains, are released to the extracellular matrix where they can bind to more extracellular matrix ligands. The shedding can have multiple effects on cell signaling. One, they rapidly downregulate signal transduction at the cell surface. Two, they can quench ligands and make them unavailable to the remaining syndecans on the cell surface. Three, they can initiate more signaling pathways as circulating soluble effectors, independent of cell surface signaling. The increase in the levels of soluble syndecans-1 and syndecan-4 in the dermal wound fluid and high levels of syndecan-3 in the serum during rheumatoid arthritis have been observed (41, 71). Early studies showed that MMP-7 mediated syndecan-1 shedding to alveolar epithelium resulted in a stable CXCL1 chemokine gradient to promote neutrophil migration (44, 72, 73). While this may depict syndecan-1 shedding as pro-inflammatory, it however allows a tight control of inflammation. The gradient formation restricts neutrophil migration at the site of epithelial injury, thus preventing further tissue damage (73). The CXCL1 secretion levels are reduced in syndecan-1 or MMP-7 knockout animals, resulting in reduced neutrophil migration (72). In addition to CXCL1, shed syndecan is known to bind with chemokines, such as CCL7, CCL11, and CCL17, minimizing the Th2 cell recruitment to the lungs (74). More recent studies showed that syndecan-1 shedding is essential for the resolution of inflammation, likely by removing the sequestered CXC chemokines (75). It is also known that soluble syndecan-1 can reduce the expression of pro-inflammatory cytokines. For example, the administration of exosomes containing syndecan-1 can be used to mitigate the expression of pro-inflammatory cytokines (e.g., IL-1β, IL-6, TNFα) and alleviate LPS induced acute lung edema and inflammation (76). While these results show that syndecan-1 shedding is required to alleviate or control inflammatory response, the opposite observations were also made. For instance, intestinal epithelial cells expressing shedding resistant syndecan-1 appears to be less prone to inflammatory damage. These experiments showed reduced neutrophil migration and TNFα expression (72). A possibility worth considering is that the transient expression of shedding resistant syndecan may have resulted in very high levels of cell bound syndecan-1, leading to the activation of other signaling pathways. Similarly, hemorrhagic shock increases systemic shedding and a decrease in the pulmonary presence of syndecan-1, leading to increased inflammation (77). In light of the observations that soluble syndecan-1 is elevated under inflammatory conditions, it has been proposed that syndecan-1 serum levels can be used as a biomarker for inflammation (78, 79). The increase in soluble syndecan-1 may occur in the later stages of inflammation. Therefore, the effectiveness of using soluble syndecan-1 as a marker in early inflammation will need further study and more effective detection tools. On the other hand, the presence of soluble syndecan-1 may be used as a marker for the severity of diseases, such as neuromyelitis optica or inflammatory breast cancers (27, 80). A final aspect of inflammation that may have been associated with syndecan shedding is angiogenesis. Inflammation promotes angiogenesis, which further enhances chronic inflammation. In other words, inflammation and angiogenesis are mutually dependent (81, 82). Previous reports showed that syndecan-1 knockout mice have increased angiogenesis due to an increase in leukocyte adhesion (83). Similarly, soluble syndecan-1 ectodomain alters angiogenesis in syndecan-1 overexpressing mice. However, this may be due to the excess proteolytic activity resulted from the modulation of proteolytic enzymes by heparan sulfate chains on the shed syndecan-1 ectodomain (84). Previous reports shows that syndecan-2 signaling is essential for angiogenesis during development, while the shed syndecan-2 is known to inhibit angiogenesis (17, 85). Syndecan-4 ectodomain shedding is associated with both acute and chronic inflammation as well as angiogenesis. One report showed that pro-inflammatory molecule LPS induces the shedding of syndecan-4 from the heart, possibly as a result of ADAMTS1, ADAMTS4, or MMP9 (61). The syndecan-4 shedding during chronic inflammation caused by diabetes mellitus resulted in an impaired macrovascular angiogenesis (86). The same report showed that treating HUVEC cells with advanced glycation end products (AGEs) leads to syndecan-4 shedding. AGEs are the harmful compounds formed by sugar and protein or fat in the blood that are typically high during diabetes mellitus. These compounds are known to enhance the expression and function of pro-inflammatory cytokine TNFα, which can also promote syndecan shedding. The TNFα mediated shedding of syndecan-4 could well be the reason for the defective angiogenesis during inflammation caused by diabetes mellitus (86).

## Perspectives and Conclusions

The current findings conclude that, irrespective of the type, syndecans are elevated during inflammation. Independent studies have indicated that syndecans can act as both pro-inflammatory and anti-inflammatory molecules, with cell surface and soluble syndecans having distinct roles. The elevation in syndecan expression is required for initial leukocyte rolling and recruitment of chemokines. However, this needs to be limited as the signaling progresses. At this point, the expression of syndecans on the cell surface may have to be controlled, which could be achieved by shedding of syndecans. The shedding of syndecans can not only reduce the signaling from cell surface, but also create a stable chemokine gradient that directs the migration of leukocytes only to the vicinity of inflammation. These limiting steps may be crucial later on, in helping the clearance of inflammation. Even though the role of syndecans in inflammation is well-studied, there are several aspects that need further investigation. Previous reports have shown that pathogens exploit GAG chains on syndecans to infect the host (87, 88). However, there is no data supporting the idea that syndecans can recognize the inflammation inducers. This is an area worth exploring in the future. In addition, the impacts of syndecan-mediated calcium regulation and matrix turnover in inflammation is still not clear and requires further studies. Finally, syndecans may influence the expression of each other, which warrants further investigation to determine how the balancing of syndecans may help to regain tissue homeostasis during inflammation. Moreover, the temporal expression levels and the rate of shedding of syndecans during different stages of inflammation is unknown. Therefore, more studies need to be done in order to establish the potential use of syndecans as biomarkers of inflammation.

## Author Contributions

SG collected the data and compiled the review.

### Conflict of Interest

The author declares that the research was conducted in the absence of any commercial or financial relationships that could be construed as a potential conflict of interest.
